# Cellular Anti-Apoptotic Effects of Dapagliflozin in Methotrexate-Induced Liver Toxicity: Bax/Bcl-2/Cyt-C/Cas-9/Cas-3 Signaling Pathway

**DOI:** 10.3390/ijms27052110

**Published:** 2026-02-24

**Authors:** Emine Sarman, Halil Asci

**Affiliations:** 1 Department of Histology and Embryology, Faculty of Medicine, Afyonkarahisar Health Sciences University, Afyonkarahisar 03030, Türkiye; 2 Department of Pharmacology, Faculty of Medicine, Suleyman Demirel University, Isparta 32260, Türkiye; halilasci@sdu.edu.tr

**Keywords:** acute liver injury, NF-κB, apoptosis, VEGF, sodium–glucose cotransporter 2 inhibitor

## Abstract

Methotrexate (MTX), an effective immunosuppressive and antiproliferative agent, is clinically restricted by its hepatotoxic potential through oxidative stress, inflammation, and apoptosis. Dapagliflozin (DAPA), a sodium–glucose cotransporter 2 inhibitor, exhibits antioxidant and anti-inflammatory actions. This study investigated the hepatoprotective effects of DAPA against MTX-induced acute liver injury. Thirty-two female Wistar albino rats were divided into four groups (*n* = 8): Control, MTX (20 mg/kg), MTX + DAPA (MTX + DAPA 10 mg/kg/day for 10 days), and DAPA. Liver samples were examined histologically, immunohistochemically (Nuclear factor NF-kappa-B p65 subunit (NF-κB p65), Tumor necrosis factor alpha (TNF-α), Interleukin 1 beta (IL-1β), Caspase (Cas)-3, Vascular endothelial growth factor (VEGF)), molecularly (Reverse transcription–polymerase chain for Bcl-2-associated X protein (Bax), B-cell lymphoma 2 (Bcl-2), Cytochrome C (Cyt-C), Apoptotic peptidase activating factor 1 (Apaf-1), Cas-9, Cas-3, Cas-12), and biochemically (total oxidant status (TOS), total antioxidant status (TAS) and oxidative stress index (OSI)). MTX induced severe hepatic injury with congestion, sinusoidal dilatation, and inflammatory infiltration, accompanied by upregulation of NF-κB, TNF-α, IL-1β, Bax, Cyt-C, Apaf-1, Cas-9, Cas-3, and Cas-12 and reduced Bcl-2. DAPA co-treatment significantly restored hepatic structure, suppressed inflammatory and apoptotic markers, and normalized VEGF expression, indicating reduced pathological angiogenesis. Although DAPA did not fully reverse MTX-induced weight loss, it effectively mitigated hepatocellular damage. DAPA protects against MTX-induced liver injury by inhibiting NF-κB/TNF-α/IL-1β-mediated inflammation, modulating Bax/Bcl-2–Cyt-C–Cas-dependent apoptosis, and balancing VEGF-driven angiogenesis. DAPA may thus serve as a promising hepatoprotective adjunct in MTX therapy.

## 1. Introduction

Methotrexate (MTX) is an effective drug widely used in the treatment of rheumatic diseases, certain cancers, and autoimmune disorders owing to its antimetabolite and immunosuppressive properties [1]. However, prolonged or high-dose administration of MTX can cause toxic effects, particularly in the liver, resulting in inflammation, oxidative stress, and tissue damage [2]. The hepatotoxic potential of MTX primarily stems from the inhibition of folate metabolism, which disrupts intracellular methylation reactions and reduces nicotinamide adenine dinucleotide phosphate production [3]. This biochemical imbalance leads to the excessive accumulation of reactive oxygen species (ROS) [4]. Elevated ROS levels overwhelm antioxidant defense systems, promoting oxidative stress that initiates a cascade of subcellular dysfunctions [5].

The accumulation of ROS represents a key early event that triggers stress responses in both the mitochondria and the endoplasmic reticulum (ER) [6]. Within mitochondria, the ROS-induced oxidation of lipids and proteins alters the Bax/Bcl-2 ratio toward the pro-apoptotic Bax form, increasing membrane permeability and causing the release of Cytochrome C (Cyt-C) into the cytoplasm [7]. This event activates the Apoptotic peptidase activating factor 1 (Apaf-1)/Caspase (Cas)-9/Cas-3 apoptotic pathway, leading to mitochondria-mediated apoptosis [8]. Concurrently, ROS disrupts protein-folding processes in the ER, causing ER stress and activation of the unfolded protein response [9]. Prolonged ER stress further enhances CCAAT/enhancer-binding protein homologous protein and Cas-12 activity, amplifying apoptotic signaling. Through these mechanisms, MTX promotes both mitochondrial and ER-mediated apoptosis, intensifying hepatocellular injury [10].

ROS accumulation also activates the Nuclear factor NF kappa B (NF-κB) signaling pathway, which increases the expression of pro-inflammatory cytokines such as Tumor necrosis factor alpha (TNF-α), and interleukin-1β (IL-1β) and interleukin-6 (IL-6). These cytokines exacerbate mitochondrial dysfunction and promote additional ROS production, forming a self-perpetuating oxidative stress–inflammation–apoptosis cycle [11]. As hepatocellular injury advances, local hypoxia develops, inducing hypoxia-inducible factor-1α expression and subsequent upregulation of Vascular endothelial growth factor (VEGF) [12]. While VEGF-driven angiogenesis initially serves as a regenerative response, excessive and dysregulated VEGF expression results in abnormal neovascularization and pathological fibrosis, ultimately contributing to irreversible hepatic dysfunction [13].

**Figure 1:**
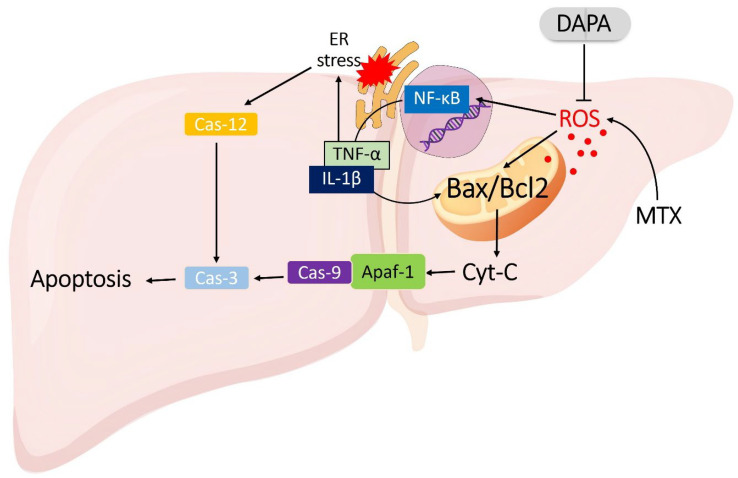
Proposed mechanism of DAPA-mediated protection against MTX-induced hepatic injury. MTX enhances ROS generation, which activates the NF-κB pathway and upregulates pro-inflammatory cytokines (TNF-α, IL-1β). The resulting inflammatory milieu promotes Bax activation and mitochondrial outer-membrane permeabilization, leading to Cyt-C release and apoptosome formation (Apaf-1/Cas-9/Cas-3), thereby triggering apoptosis. Concurrently, excessive ROS and cytokine signaling provoke ER stress, activating Cas-12 and further amplifying apoptotic signaling. DAPA attenuates these pathological processes by suppressing ROS generation, inhibiting NF-κB activation, and re-balancing Bax/Bcl-2 expression, thus limiting mitochondrial and ER-mediated apoptosis. Overall, DAPA mitigates oxidative, inflammatory, and apoptotic stress, preserving hepatic cellular integrity. MTX: Methotrexate, DAPA: Dapagliflozin, Apaf-1: Apoptotic peptidase activating factor 1, Cas-9: Caspase 9, Cas-3: Caspase 3, Cas-12: Caspase 12, Bax: Bcl-2-associated X protein, Bcl2: B-cell lymphoma 2, Cyt-C: Cytochrome C, ROS: reactive oxygen species, ER: endoplasmic reticulum, NF-κB: Nuclear factor NF-kappa-B, IL-1β: Interleukin 1 beta, TNF-α: Tumor necrosis factor alpha, VEGF: Vascular endothelial growth factor.

In recent years, Dapagliflozin (DAPA), a sodium–glucose cotransporter-2 (SGLT-2) inhibitor, has drawn attention not only for its antidiabetic effects but also for its antioxidant, anti-inflammatory, and anti-apoptotic properties [14]. Previous studies have demonstrated that DAPA reduces ROS formation, inhibits NF-κB activation, and suppresses the overproduction of pro-inflammatory cytokines [15]. By preserving mitochondrial function and supporting cellular energy homeostasis, DAPA may counteract the cascade of oxidative stress and apoptotic signaling triggered by MTX. Hence, DAPA could potentially disrupt the vicious cycle of oxidative and inflammatory injury, thereby protecting hepatocyte integrity and reducing fibrosis development [16,17]. This study aims to investigate the hepatoprotective effects of DAPA in an experimental model of MTX-induced acute liver injury, focusing on its anti-inflammatory and anti-apoptotic mechanisms (Figure 1). The findings are expected to provide new insights into the pathophysiological basis of MTX-induced hepatotoxicity and the potential therapeutic role of DAPA in liver protection.

## 2. Results

### 2.1. Histopathologic Examination

In the liver tissue sections of the control and DAPA groups, it was observed that hepatocyte arrangement was preserved, no pathologic findings were observed around the portal areas and central vein, and the general histologic architecture was largely preserved. On the other hand, degenerative changes in hepatocytes and hepatocytes with eosinophilic cytoplasm marked congestion and dilatation in the central vein and mononuclear cell infiltration were observed in the MTX group.

In the MTX + DAPA group, histopathologic changes in liver tissue were significantly reduced compared to the MTX group. In this group, structural integrity of hepatocytes was largely preserved, central vein congestion and mononuclear cell infiltration were mild, and degenerative changes in hepatocytes were reduced.

**Figure 2:**
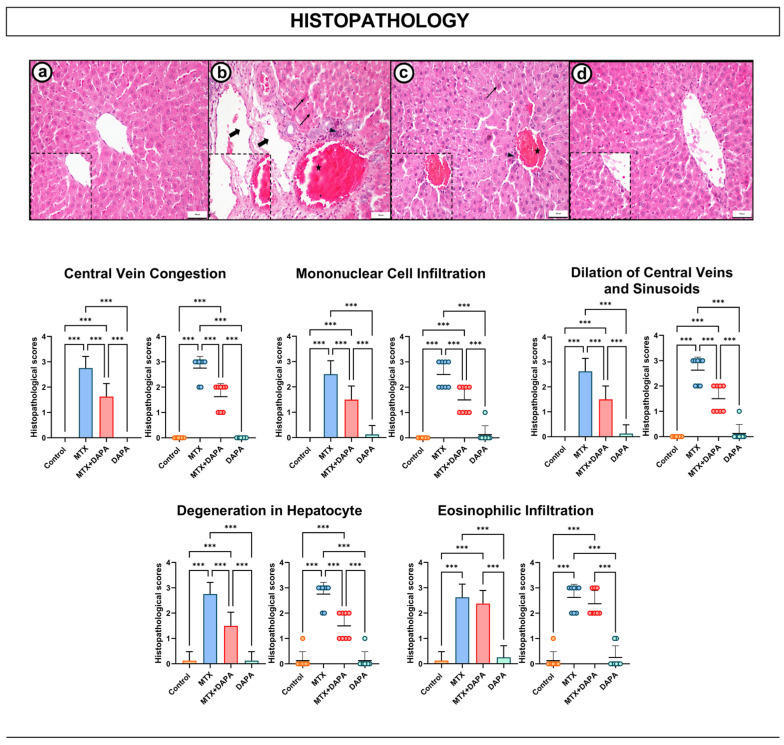
Histopathological reactivities in the liver tissues. HE-stained images of liver tissue sections of all groups. Liver histology was normal in the control group (**a**). In the MTX group, hepatocyte degeneration (arrow), and eosinophilic infiltration (arrow), marked congestion (asterisk), dilatation (thick arrow) and mononuclear cell infiltration (arrowhead) in the central vein (**b**). In the MTX + DAPA group, these pathologic findings were attenuated: congestion (arrowhead), mononuclear cell infiltration (arrowhead) and hepatocyte degeneration (arrow) were reduced (**c**). Normal liver histology is seen in the DAPA group (**d**). 20×; scale bar, 100 μm. Quantitative assessment of histopathological parameters revealed a significant reduction in the MTX group compared to control, while DAPA treatment markedly improved hepatocyte integrity. Data are shown as mean ± SD (bar graph) and individual animal values (dot plot). Statistical evaluation was performed using one-way ANOVA followed by Tukey’s post hoc test (*** *p* < 0.001). HE: Hematoxylin and Eosin, MTX: Methotrexate, DAPA: Dapagliflozin.

These findings support that DAPA may play a protective role against MTX-induced liver toxicity at the tissue level (Figure 2).

### 2.2. Inflammatory Immunoreactivity Findings

Evaluation of inflammatory markers revealed distinct expression patterns among the experimental groups. TNF-α immunoreactivity was low or negative in the control and DAPA groups, whereas strong expression was detected in the MTX group, particularly around the central vein and within hepatocytes. In the MTX + DAPA group, TNF-α expression was markedly reduced, suggesting that DAPA may exert a hepatoprotective effect by suppressing MTX-induced inflammation.

Similarly, NF-κB p65 immunoreactivity showed low-level cytoplasmic localization in the control and DAPA groups, while a marked increase—predominantly nuclear—was observed in the MTX group. In the MTX + DAPA group, NF-κB p65 immunoreactivity decreased, and nuclear staining was partially diminished. These findings indicate that DAPA may attenuate MTX-induced inflammatory activation by inhibiting the NF-κB signaling pathway.

**Figure 3:**
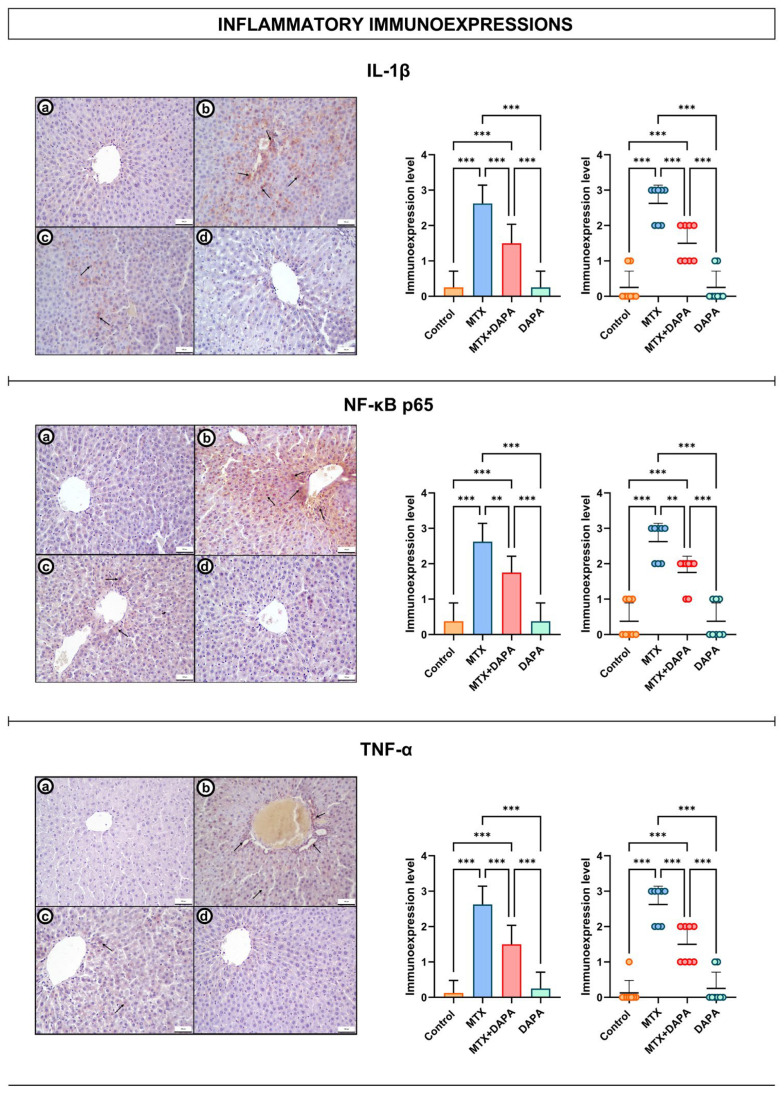
NF-κB p65, IL-1β and TNF-α immunoreactivities in the liver tissues. Low levels of immunoreactivity were observed in the control and DAPA groups (**a**,**d**). In the MTX group, NF-κB p65 was prominently localized within the nuclei and both TNF-α and IL-1β showed markedly increased expression in hepatocytes and inflammatory cells (**b**, arrows). In the MTX + DAPA group, nuclear staining and overall expression of these markers were reduced, indicating attenuation of the inflammatory response (**c**, arrows). DAPA administration exerted a protective effect on the liver by suppressing MTX-induced inflammation. 20×; scale bar, 100 μm. Quantitative analysis of NF-κB p65, IL-1β and TNF-α immunoexpressions revealed a significant upregulation in the MTX group relative to control, which was markedly suppressed by DAPA treatment. Data are expressed as mean ± SD (bar graph) and individual animal values (dot plot). Statistical comparisons were performed using one-way ANOVA followed by Tukey’s post hoc test (** *p* < 0.01, *** *p* < 0.001). MTX: Methotrexate, DAPA: Dapagliflozin, NF-κB p65: Nuclear factor NF-kappa-B p65 subunit, IL-1β: Interleukin 1 beta, TNF-α: Tumor necrosis factor alpha.

IL-1β immunoreactivity was weak or negative in the control and DAPA groups but significantly increased in hepatocytes and inflammatory cells of the MTX group. In the MTX + DAPA group, IL-1β expression was decreased, and the overall inflammatory response was attenuated, supporting the anti-inflammatory potential of DAPA against MTX-induced hepatic injury (Figure 3).

### 2.3. Angiogenetic and Apoptotic Immunoreactivity Findings

**Figure 4:**
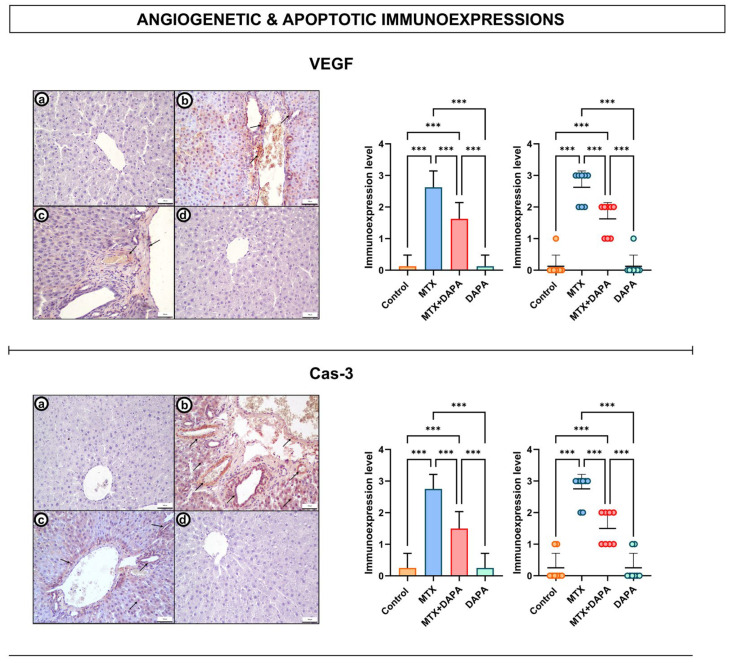
Cas-3 and VEGF immunoreactivities in the liver tissues. In the control group, negative or very mild Cas-3 and VEGF immunoreactivities were observed (**a**,**d**). In the MTX group, prominent Cas-3 positivity (arrow) was observed around the central vein and hepatocytes and intense VEGF immunoreactivity (arrow) was observed in the periportal and central vein regions (**b**). In the MTX + DAPA group, moderate or mild immunoreactivity was detected for both markers (arrow) (**c**). Negative or very mild immunoreactivity levels were maintained in the DAPA group. These findings suggest that DAPA shows a protective effect in liver tissue by suppressing MTX-induced apoptosis and angiogenesis. 20×; scale bar, 100 μm. Quantitative analysis of Cas-3 and VEGF immunoexpressions revealed a significant upregulation in the MTX group relative to control, which was markedly suppressed by DAPA treatment. Data are expressed as mean ± SD (bar graph) and individual animal values (dot plot). Statistical comparisons were performed using one-way ANOVA followed by Tukey’s post hoc test (*** *p* < 0.001). MTX: Methotrexate, DAPA: Dapagliflozin, Cas-3: Caspase 3, VEGF: Vascular endothelial growth factor.

Evaluation of angiogenesis (VEGF)- and apoptosis (Cas-3)-related markers revealed distinct differences among the groups. VEGF expression was negative or very weak in the control and DAPA groups. In the MTX group, VEGF immunoreactivity was markedly increased, especially in periportal regions, reflecting the angiogenic response secondary to liver injury. In contrast, VEGF expression in the MTX + DAPA group was decreased and remained at a lower level than that in the MTX group (Figure 4).

Similarly, Cas-3 expression was negative or very weak in the control and DAPA groups, whereas strong immunoreactivity was detected in the MTX group, particularly in hepatocytes surrounding the central vein. Notably, Cas-3 expression was reduced in the MTX + DAPA group compared with the MTX group.

### 2.4. Apoptotic Gene Findings

**Figure 5:**
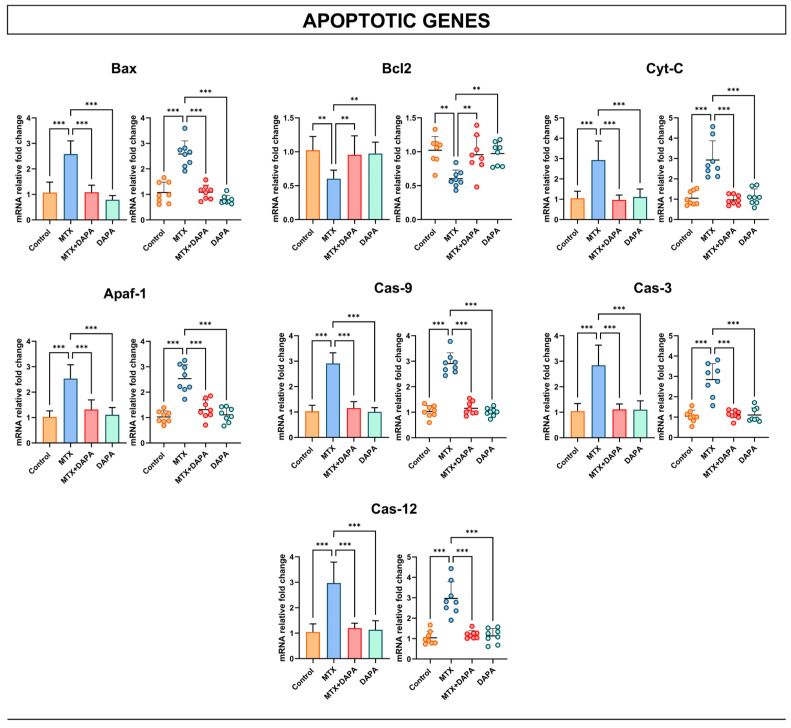
Apoptotic gene activities in the liver tissues. Quantitative analysis of Bax, Cyt-C, Apaf-1, Cas-9, Cas-3, and Cas-12 immunoexpressions revealed a significant upregulation in the MTX group relative to the control, which was markedly suppressed by DAPA treatment. In contrast, the anti-apoptotic Bcl-2 expression was significantly decreased in the MTX group but restored to near-control levels following DAPA administration. Data are expressed as mean ± SD (bar graph) and individual animal values (dot plot). Statistical comparisons were performed using one-way ANOVA followed by Tukey’s post hoc test (** *p* < 0.01, *** *p* < 0.001). MTX: Methotrexate, DAPA: Dapagliflozin, Apaf-1: Apoptotic peptidase activating factor 1, Cas-9: Caspase 9, Cas-3: Caspase 3, Cas-12: Caspase 12, Bax: Bcl-2-associated X protein, Bcl2: B-cell lymphoma 2, Cyt-C: Cytochrome C.

Analysis of apoptotic gene expression revealed a marked upregulation of Bax, Cyt-C, Apaf-1, Cas-9, Cas-3 and Cas-12 in the MTX group compared with the control and DAPA-only groups. In contrast, the MTX + DAPA group exhibited a significant reduction in the expression of these pro-apoptotic genes, indicating that DAPA mitigated MTX-induced apoptotic activation. Conversely, the anti-apoptotic Bcl-2 gene showed an opposite trend: its expression was markedly decreased in the MTX group compared with the control and DAPA groups, whereas a significant elevation was observed in the MTX + DAPA group (Figure 5).

These results indicate that DAPA significantly modulates apoptotic gene expression, leading to a marked decrease in pro-apoptotic and an increase in anti-apoptotic markers compared with MTX-treated rats.

### 2.5. Biochemical Analysis Findings

**Figure 6:**
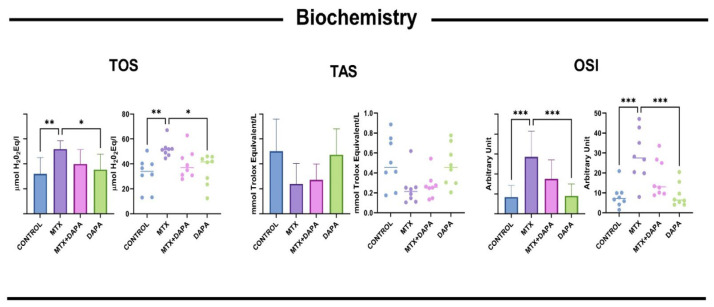
TAS, TOS, and OSI values in liver tissue. TOS: total oxidant status (µmol H_2_O_2_ equivalent/L), TAS: total antioxidant status (mmol Trolox equivalent/L), OSI: oxidative stress index, MTX: Methotrexate, DAPA: Dapagliflozin, * *p*  <  0.05, ** *p*  <  0.01, *** *p*  <  0.001.

Significant differences were observed in oxidative stress markers between the MTX and control groups. The control group maintained low TOS and OSI values and high TAS values. The antioxidant defense system was depleted in response to MTX-induced oxidative stress. Furthermore, TOS and OSI values in the MTX group were significantly higher than those in the control and DAPA groups (*p* < 0.001), indicating increased oxidative damage (Figure 6).

### 2.6. Animal Weight Measurement

**Figure 7:**
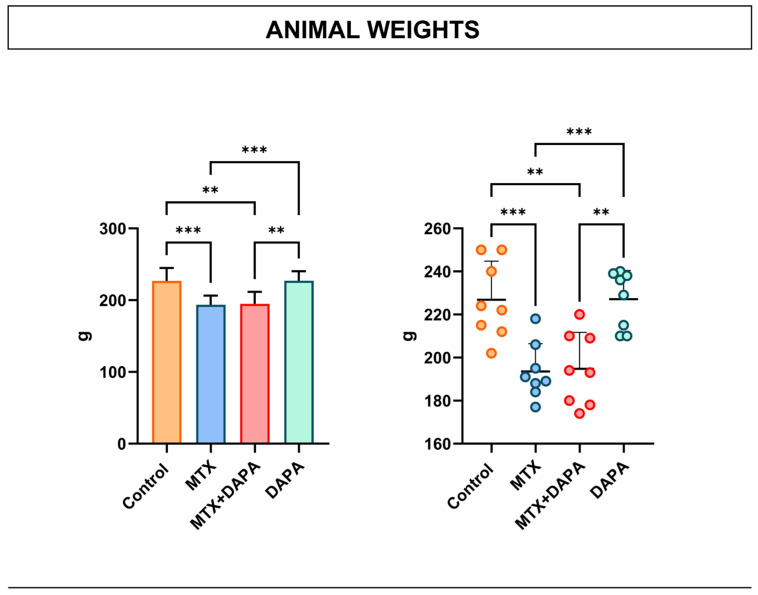
Quantitative scoring of animal weights. Quantitative analysis of animal weight measurement. It revealed a significant down-regulation in the MTX group compared to control but no significant change compared to DAPA treatment. Data are expressed as mean ± SD (bar graph) and individual animal values (dot plot). Statistical comparisons were performed using one-way ANOVA followed by Tukey’s post hoc test (** *p* < 0.01, *** *p* < 0.001). MTX: Methotrexate, DAPA: Dapagliflozin.

Body weight analysis showed a significant decrease in the MTX group compared to the control group and the MTX + DAPA group. In contrast, the DAPA group exhibited significantly higher body weight values compared to both the MTX and MTX + DAPA groups. These findings suggest that MTX significantly decreased body weight, whereas the DAPA-treated group maintained low weights similar to the MTX group, suggesting that DAPA had no significant ameliorative effect on this parameter (Figure 7).

The comprehensive statistical analysis of all histopathological, immunohistochemical, molecular, and physiological parameters is presented in Table 1. Intergroup comparisons revealed distinct pathological and biochemical patterns that delineate the cascade of MTX-induced hepatic injury and the multifaceted protective effects of DAPA.

## 3. Discussion

This study investigated the hepatoprotective efficacy of DAPA, a SGLT2 inhibitor with well-documented anti-inflammatory and antioxidant properties, in an MTX-induced acute liver injury model in female Wistar albino rats.

MTX, despite its efficacy as an immunosuppressive and antiproliferative agent, is clinically limited by its potential to cause multiorgan toxicity [18]. The systemic metabolic stress induced by MTX was evident from the pronounced body weight loss observed in MTX-treated rats, likely resulting from gastrointestinal toxicity, appetite suppression, and enhanced catabolic turnover [19]. DAPA co-administration failed to completely normalize body weight but attenuated the MTX-induced reduction, suggesting a partial systemic protective effect. Hence, alterations in body weight reflected both the metabolic burden of MTX and the limited systemic compensation provided by DAPA. Although DAPA demonstrated marked hepatoprotective effects against MTX-induced injury, it did not fully reverse the associated body weight loss. This finding suggests that MTX-induced weight reduction may involve systemic factors beyond liver-specific damage, such as gastrointestinal toxicity, reduced food intake, metabolic stress, or generalized inflammation. Therefore, while DAPA may exert organ-protective effects at the hepatic level through its antioxidant and anti-inflammatory properties, these mechanisms alone may not be sufficient to counteract the broader systemic consequences of MTX administration.

At the cellular level, MTX-induced hepatotoxicity represents a complex interplay of oxidative stress, inflammation, and apoptosis [6]. The inhibition of folate metabolism by MTX impairs redox homeostasis, leading to the accumulation of ROS [20,21]. Elevated ROS triggers activation of the NF-κB pathway, which promotes the transcription of pro-inflammatory cytokines such as TNF-α and IL-1β [22]. These mediators disrupt hepatocyte membrane integrity, induce sinusoidal congestion, and impair calcium homeostasis, thereby amplifying cellular injury [22,23]. In the current study, MTX exposure significantly increased hepatic NF-κB, TNF-α, and IL-1β levels, accompanied by histopathological findings including central vein congestion, sinusoidal dilatation, inflammatory infiltration, and hepatocellular degeneration—collectively indicative of an active inflammatory milieu.

DAPA treatment substantially alleviated these pathological changes. Decreased NF-κB, TNF-α, and IL-1β levels, together with marked histological improvement, demonstrate that DAPA effectively suppresses inflammatory activation and preserves hepatic microcirculation. The underlying mechanism likely involves the inhibition of NF-κB signaling and attenuation of ROS overproduction. These results align with findings by Li et al. (2024) and Hazem et al. (2022), who reported that DAPA mitigates oxidative and inflammatory responses through NF-κB inhibition and cytokine down-regulation [24,25]. In addition to direct NF-κB inhibition, Dapagliflozin may exert upstream regulatory effects through cytoprotective signaling pathways such as AMPK/SIRT1, Nrf2/HO-1, and PI3K/Akt. These pathways are known to reduce oxidative stress, suppress inflammatory activation, and limit apoptosis by improving mitochondrial function, enhancing antioxidant defenses, and promoting cell survival. Although not directly evaluated in the present study, their involvement may partly explain the broad hepatoprotective effects of DAPA observed here.

The inflammatory cascade triggered by MTX extends to apoptotic pathways mediated by mitochondrial and ER stress [26]. TNF-α signaling enhances Bax expression, increasing mitochondrial membrane permeability and promoting Cyt-C release [27]. Cyt-C interacts with Apaf-1 to form the apoptosome complex, activating Cas-9 and subsequently Cas-3, culminating in apoptotic cell death [28]. In parallel, the excessive production of ROS and inflammatory mediators induces ER stress and activates Cas-12, thereby amplifying the apoptotic signal [29]. Conversely, Bcl2 stabilizes mitochondrial membranes and counteracts Cyt-C release [30]. In this study, MTX administration markedly upregulated Bax, Cyt-C, Apaf-1, Cas-9, Cas-3, and Cas-12 expression while reducing Bcl2 levels, indicating concurrent activation of mitochondrial and ER-mediated apoptosis. DAPA co-treatment significantly reversed these alterations, restoring Bcl2 expression and suppressing pro-apoptotic gene activation. These results indicate that DAPA effectively protects hepatocytes by inhibiting dual apoptotic pathways, thereby preserving cellular integrity.

The progression of MTX-induced inflammation and apoptosis was accompanied by increased VEGF expression, which likely represents a compensatory angiogenic response to tissue injury [31]. However, sustained VEGF overexpression may lead to maladaptive neovascularization [32]. DAPA administration normalized VEGF levels, suggesting that mitigation of inflammatory and apoptotic stress concurrently restrains aberrant angiogenesis. Consistent with injury-linked angiogenic modulation, DAPA has been shown to attenuate VEGF-related signaling in an ethanol-induced gastric injury model [33], aligning with our observation of normalized VEGF levels under reduced inflammatory/oxidative stress [34]. Although DAPA demonstrated marked hepatoprotective effects in the present experimental model, rare cases of Dapagliflozin-associated hepatotoxicity and idiosyncratic drug-induced liver injury have been reported in clinical settings, particularly in patients with pre-existing liver disease. These events are considered patient-specific and multifactorial rather than reflecting a direct hepatotoxic pharmacological mechanism. Therefore, our findings should be interpreted within the controlled conditions of an experimental model. While DAPA exhibits cytoprotective effects at the tissue level, careful clinical monitoring remains important, especially in individuals with underlying hepatic dysfunction.

Future studies investigating the involvement of Nrf2/HO-1, AMPK, and SIRT1 signaling pathways are warranted to further clarify the molecular mechanisms underlying the hepatoprotective effects of DAPA.

### Limitations

This study has certain limitations. First, it focused solely on acute MTX-induced hepatotoxicity, and, thus, long-term or cumulative protective effects of DAPA under chronic exposure remain uncertain. The single-dose, fixed-duration design did not allow for assessment of dose-dependent pharmacodynamics. The exclusive use of female rats limits extrapolation regarding sex-specific responses. Moreover, the molecular pathways responsible for DAPA’s antioxidant actions (such as Nuclear factor erythroid 2-related factor 2 (NRF2)/Heme oxygenase 1 (HO-2), AMP-activated protein kinase (AMPK), and Sirtuin 1 (SIRT1) were not examined and warrant detailed exploration in future studies.

Another limitation of the present study is that MTX-induced pancreatic alterations were not evaluated. Although the current protocol focused primarily on hepatic injury, accumulating evidence suggests that MTX may also exert oxidative and inflammatory effects on pancreatic tissue, potentially contributing to systemic toxicity [30,31,32,33,34]. Future studies integrating biochemical and histopathological assessments of the pancreas could provide a more comprehensive view of MTX-related multiorgan injury. Finally, as an experimental animal study, the findings require cautious interpretation before translation to clinical contexts.

## 4. Materials and Methods

### 4.1. Animals and Ethical Approval

A total of 32 adult female Wistar albino rats (weighing 300–350 g) were used in the study. The animals were housed under standardized conditions, including a controlled temperature of 21 ± 2 °C, relative humidity of 55–60%, and a 12 h light/dark cycle. All rats had free access to standard laboratory chow and water throughout the experiment. Prior to the start of the study, they underwent an acclimatization period of at least 7 days. Random assignment to experimental groups was performed using a computer-generated random number sequence. All experimental procedures were conducted in accordance with institutional ethical guidelines and were approved by the Suleyman Demirel University local animal ethics committee (Approval Date: 11 July 2024, Approval No: 08/312).

### 4.2. Experimental Protocol

A total of 32 adult female Wistar albino rats were randomly divided into four experimental groups (*n* = 8 in each group). Four different experimental groups were formed in the study. In the control group, 1 mL of saline was given orally every day during the experiment and saline was also administered intraperitoneally (ip) on day 2. In the MTX group, a single dose of 20 mg/kg MTX (ip, 50 mg/mL vial, Koçak, Istanbul, Türkiye) was administered on the 2nd day of the experiment and 1 mL of saline was given ip every day for 10 days [35]. In the MTX + DAPA group, a single dose of 20 mg/kg MTX ip was administered on day 2, and in addition, 10 mg/kg DAPA (1 mL, Forziga, AstraZeneca, Istanbul, Türkiye) was administered orally every day for 10 days [36]. Finally, 10 mg/kg DAPA was administered to the DAPA group orally every day for 10 days and saline was injected ip on the 2nd day of the experiment.

All rats were anesthetized with 90 mg/kg Ketamine (Keta-Control, Doğa İlaç, Istanbul, Türkiye) and 8–10 mg/kg Xylazin (XylazinBio, Bioveta, Ivanovice na Hané, Czech Republic) 24 h after the last drug administration. Following anesthesia, the rats were euthanized by surgical exsanguination method by taking the blood from the inferior vena cava following abdominal incision. Liver tissues of the rats were removed and half of them were placed in eppendorfs in a cold environment and to be stored at −80 °C for genetic analysis. In the other half of the tissues, which were placed in 10% formaldehyde solution, histopathologic Hematoxylin–Eosin (HE) staining (hepatocyte degeneration, eosinophilic hepatocytes, congestion, dilatation and mononuclear cell infiltration were evaluated), inflammatory and apoptotic parameters such as NF-κB, TNF-α and VEGF levels were evaluated immunohistochemically. The PCR method was used to examine Apaf-1, Cas-9, Cas-3, Cas-12, Bax, Bcl2 and Cyt-C gene expressions from apoptotic intracellular pathways.

### 4.3. Reverse Transcription–Polymerase Chain Reaction (RT-qPCR)

Using the manufacturer’s protocol, RNA was isolated from homogenized tissues with the GeneAll RiboEx (TM) RNA Isolation Kit (GeneAll Biotechnology, Seoul, Republic of Korea). The amount and purity of the RNAs obtained were measured with the BioSpec-nano nanodrop (Shimadzu Ltd., Kyoto, Japan) device and 1 µg RNA was used for cDNA synthesis. cDNA synthesis with the A.B.T.™ cDNA Synthesis Kit (Atlas Biotechnology, Ankara, Turkey) was carried out in a thermal cycler according to the protocol. Primer designs were made by detecting specific mRNA sequences and testing possible primer sequences using the NCBI website. The sequences of the primer sequences used are shown in Table 2. Expression levels of genes were measured in a Biorad CFX96 (Hercules, CA, USA) real-time PCR instrument using 2X SYBR green master mix (Nepenthe, Istanbul, Turkey). In the study, the GAPDH gene was used as a housekeeping gene. The reaction mixture was prepared according to the manufacturer’s protocol to a final volume of 20 µL. The resulting reaction mixture was placed in a real-time qPCR device determined according to the kit manufacturer’s protocol, and each sample was studied in 3 replications. PCR conditions, initial denaturation 94 °C 10 min. Denaturation at 95 °C for 15 s was carried out for 1 cycle and annealing/extension at 57 °C for 30 s was applied for 40 cycles. Relative mRNA levels were calculated by applying the 2−ΔΔCt formula to the normalized results.

### 4.4. Histochemical Analysis

After the experimental animals were sacrificed, the liver tissues were fixed with 10% neutral buffered formaldehyde for 24 h. After fixation, they were washed under running water for 24 h. They were passed through a series of rinsing alcohols and made transparent in xylol. Sections that were 5 µ thick were taken from the tissues embedded in hard paraffin. The sections were stained with HE. They were evaluated and photographed with a photomicroscope (Eclipse E-600 Nikon, Tokyo, Japan) and image analysis system (NIS Elements Nikon, Tokyo, Japan). Histopathologic changes such as degeneration of hepatocytes, eosinophilic hepatocytes, marked congestion, dilatation and mononuclear cell infiltration in the central vein were evaluated semi-quantitatively. A scoring system based on the severity and distribution of findings is presented in Table 3. In each animal, 10 randomly selected areas were examined under a light microscope at 40× magnification [37,38].

### 4.5. Immunohistochemical Analysis

After 5 µ thick sections were taken from the paraffin blocks, they were deparaffinized and passed through alcohol series and boiled in citrate buffer solution for 20 min. After 5 min treatment with H_2_O_2_ (TA-060-HP, Lab Vision Corporation, Fremont, CA, USA) and Ultra V Block (TA-125-UB, Thermo Scientific, Cheshire, UK) solution, they were incubated with Caspase-3 (sc-65497, Santa Cruz, CA, USA, 1:200), VEGF (sc-7269, Santa Cruz, CA, USA, 1:200), TNF alpha (sc-52746, Santa Cruz, CA, USA, 1:200), NFκB p65 (sc-8008, Santa Cruz, CA, USA, 1:200), and IL-1β (sc-52012, Santa Cruz, CA, USA, 1:200) for 60 min. Then they were incubated with secondary antibody (Biotinylated Goat Anti-Polyvalent TP-125-BN, Thermo Scientific, Cheshire, UK) and Strepavidin HRP (Horse radish peroxidase) (TS-125-HR, Thermo Scientific, Cheshire, UK) for 30 min at room temperature. After DAB (3,3′-diaminobenzidine) (TA-125 HD, Thermo Scientific, Cheshire, UK) solution, tissues were counter-stained with Mayer’s Hematoxylin and covered with Entellan. All preparations were evaluated and photographed using an Eclipse E-600 Nikon, Japan photomicroscope and image analysis system (NIS Elements Nikon, Tokyo, Japan). Immuno-staining intensity was evaluated semi-quantitatively on a 0–3 scale in ten fields, as described in Table 3. For immunohistochemical examination, preparations were analyzed independently for each antibody [39].

### 4.6. Biochemical Analysis

To assess oxidative and antioxidative characteristics, liver tissue samples were homogenized. Using Rel Assay Diagnostics kits (Gaziantep, Turkey) and a Beckman Coulter AU 5800 apparatus (Beckman Coulter, Brea, CA, USA), TAS (mmol Trolox equivalent/L) and TOS (µmol H_2_O_2_ equivalent/L) were measured spectrophotometrically. TOS was divided by TAS to determine OSI [40]. The TAS method involves reducing the dark blue–green ABTS radical to a colorless form by antioxidants, with absorbance changes at 660 nm reflecting antioxidant levels. Results are expressed in millimolar Trolox equivalents per gram of protein. The results are normalized against hydrogen peroxide and reported as micromolar hydrogen peroxide equivalents per gram of protein [41].

### 4.7. Statistical Analysis

All data were analyzed using GraphPad Prism version 9.3. Results are expressed as mean ± standard deviation (SD). Comparisons among groups were performed using one-way analysis of variance (ANOVA) followed by Tukey’s post hoc test for multiple comparisons. A *p*-value < 0.05 was considered statistically significant.

## 5. Conclusions

In summary, MTX-induced hepatic injury is orchestrated through a triad of inflammation, apoptosis, and compensatory angiogenesis. DAPA counteracts this triad by suppressing NF-κB/TNF-α/IL-1β-mediated inflammation, normalizing the Bax/Bcl-2/Cyt-C/Cas axis to limit apoptosis, and regulating VEGF expression to restore vascular homeostasis. The potential modulation of Nrf2/HO-1 and AMPK/SIRT1 pathways further enhances DAPA’s cytoprotective repertoire. Collectively, these findings establish DAPA as a promising hepatoprotective agent capable of mitigating MTX-induced toxicity. Given the expanding clinical application of SGLT2 inhibitors beyond glycemic control, DAPA’s demonstrated efficacy in preserving hepatic structure and function highlights its translational potential as an adjunctive therapy in patients requiring long-term MTX administration.

## Figures and Tables

**Table 1:** Comparison of *p*-values between groups.

Individual *p*-Value (Tukey)	Control vs. MTX	Control vs. MTX + DAPA	Control vs. DAPA	MTX vs. MTX + DAPA	MTX vs. DAPA	MTX + DAPA vs. DAPA
Histopathology						
Degeneration in hepatocyte	<0.001	<0.001	>0.999	<0.001	<0.001	<0.001
Eosinophilic Hepatocytes	<0.001	<0.001	=0.950	=0.711	<0.001	<0.001
Central Vein Congestion	<0.001	<0.001	>0.999	<0.001	<0.001	<0.001
Mononuclear Cell Infiltrate	<0.001	<0.001	=0.931	<0.001	<0.001	<0.001
Dilation of Central Veins and Sinusoids	<0.001	<0.001	=0.929	<0.001	<0.001	<0.001
Immunohistochemistry						
NF-κB p65	<0.001	<0.001	>0.999	=0.009	<0.001	<0.001
IL-1β	<0.001	<0.001	>0.999	<0.001	<0.001	<0.001
TNF-α	<0.001	<0.001	=0.951	<0.001	<0.001	<0.001
VEGF	<0.001	<0.001	>0.999	<0.001	<0.001	<0.001
Cas-3	<0.001	<0.001	>0.999	<0.001	<0.001	<0.001
Apoptotic Genes						
Bax	<0.001	>0.999	=0.437	<0.001	<0.001	=0.403
Bcl2	=0.002	=0.918	=0.963	=0.008	=0.006	=0.998
Cyt-C	<0.001	=0.991	=0.996	<0.001	<0.001	=0.955
Apaf-1	<0.001	=0.432	=0.970	<0.001	<0.001	=0.700
Cas-9	<0.001	=0.805	=0.999	<0.001	<0.001	=0.722
Cas-3	<0.001	=0.990	=0.993	<0.001	<0.001	>0.999
Cas-12	<0.001	=0.916	=0.981	<0.001	<0.001	<0.993
Biochemistry						
TOS	=0.007	=0.504	=0.927	=0.166	=0.032	=0.856
TAS	=0.53	=0.109	=0.992	=0.985	=0.096	=0.186
OSI	<0.001	=0.202	=0.999	=0.091	<0.001	=0.257
Animal Weight						
	<0.001	=0.001	>0.999	=0.998	<0.001	=0.001

Data are expressed as mean ± SD (n = 8 per group). One-way ANOVA followed by Tukey’s post hoc test was used for statistical comparisons. MTX: Methotrexate, DAPA: Dapagliflozin, Apaf-1: Apoptotic peptidase activating factor 1, Cas-9: Caspase 9, Cas-3: Caspase 3, Cas-12: Caspase 12, Bax: Bcl-2-associated X protein, Bcl2: B-cell lymphoma 2, Cyt-C: Cytochrome C, NF-κB: Nuclear factor NF-kappa-B, IL-1β: Interleukin 1 beta, TNF-α: Tumor necrosis factor alpha, VEGF: Vascular endothelial growth factor.

**Table 2:** Primary sequences, product size and accession numbers of genes.

Genes	Primer Sequence	Product Size	Accession Number
GAPDH (HouseKeeping)	F: AGTGCCAGCCTCGTCTCATA	248 bp	NM_017008.4
	R: GATGGTGATGGGTTTCCCGT		
Apaf-1	F: GAGCTGGGTAGACGGCTTTC	212 bp	NM_023979.2
	R: CCCGGATCCAGGACACAAAA		
Cas-9	F: AGCCAGATGCTGTCCCATAC	148 bp	XM_039110693.1
	R: CAGGAACCGCTCTTCTTGTC		
Cas-3	F: GGCCGACTTCCTGTATGCTT	110 bp	NM_001436900.1
	R: CGTACAGTTTCAGCATGGCG		
Cas-12	F: CTGCATCAGAATCCAGGGGA	212 bp	NM_130422.1
	R: TCGGCCTTCCTTCTCCATCA		
Bax	F. CACGTCTGCGGGGAGTCAC	419 bp	NM_017059.2
	R: TAGAAAAGGGCAACCACCCG		
Bcl2	F: CATCTCATGCCAAGGGGGAA	284 bp	NM_016993.2
	R: TATCCCACTCGTAGCCCCTC		
Cyt-C	F: TAAATATGAGGGTGTCGC	192 bp	NM_012839.2
	R: AAGAATAGTTCCGTCCTG		

F: forward, R: reverse, GAPDH: Glyceraldehyde-3-phosphate dehydrogenase, Apaf-1: Apoptotic peptidase activating factor 1, Cas-9: Caspase 9, Cas-3: Caspase 3, Cas-12: Caspase 12, Bax: Bcl-2-associated X protein, Bcl2: B-cell lymphoma 2, Cyt-C: Cytochrome C.

**Table 3:** Histopathological and immunohistochemical scores of liver tissues.

Scoring	Immunohistochemical	Histopathological
**0/(−)**	no change	negative
**1/(+)**	mild change	focal weak
**2/(++)**	moderate change	diffuse weak
**3/(+++)**	severe change	diffuse strong

Scoring: 0 (−), no change/negative; 1 (+), mild change/focal weak; 2 (++), moderate change/diffuse weak; 3 (+++), severe change/diffuse strong.

## Data Availability

Data will be made available on request.
