# Correction: Sarman, E.; Asci, H. Cellular Anti-Apoptotic Effects of Dapagliflozin in Methotrexate-Induced Liver Toxicity: Bax/Bcl-2/Cyt-C/Cas-9/Cas-3 Signaling Pathway. *Int. J. Mol. Sci.* 2026, *27*, 2110

**DOI:** 10.3390/ijms27073158

**Published:** 2026-03-31

**Authors:** Emine Sarman, Halil Asci

**Affiliations:** 1 Department of Histology and Embryology, Faculty of Medicine, Afyonkarahisar Health Sciences University, Afyonkarahisar 03030, Türkiye; 2 Department of Pharmacology, Faculty of Medicine, Suleyman Demirel University, Isparta 32260, Türkiye; halilasci@sdu.edu.tr

There was an error in the original publication [1]. The Statistical Analysis section was unintentionally omitted in the published version of the manuscript. A correction has been made to 4.7. The authors state that the scientific conclusions are unaffected. This correction was approved by the Academic Editor. The original publication has also been updated.

*4.7.*  *Statistical Analysis*

All data were analyzed using GraphPad Prism version 9.3. Results are expressed as mean ± standard deviation (SD). Comparisons among groups were performed using one-way analysis of variance (ANOVA) followed by Tukey’s post hoc test for multiple comparisons. A *p*-value < 0.05 was considered statistically significant.

## References

[1] Sarman E., Asci H. (2026). Cellular Anti-Apoptotic Effects of Dapagliflozin in Methotrexate-Induced Liver Toxicity: Bax/Bcl-2/Cyt-C/Cas-9/Cas-3 Signaling Pathway. Int. J. Mol. Sci..

